# Mutations in *Podospora anserina MCM1* and *VelC* Trigger Spontaneous Development of Barren Fruiting Bodies

**DOI:** 10.3390/jof10010079

**Published:** 2024-01-19

**Authors:** Insaf Essadik, Charlie Boucher, Cécilia Bobée, Éva Cabet, Valérie Gautier, Hervé Lalucque, Philippe Silar, Florence Chapeland-Leclerc, Gwenaël Ruprich-Robert

**Affiliations:** Université Paris Cité, CNRS, UMR 8236—LIED, F-75013 Paris, Francececilia.bobee@univ-paris-diderot.fr (C.B.); eva.cabet@u-paris.fr (É.C.); valerie.gautier@u-paris.fr (V.G.); herve.lalucque@u-paris.fr (H.L.); florence.leclerc@u-paris.fr (F.C.-L.)

**Keywords:** perithecia, *Podospora anserina*, development, sexual reproduction, ascohymenials

## Abstract

The ascomycete *Podospora anserina* is a heterothallic filamentous fungus found mainly on herbivore dung. It is commonly used in laboratories as a model system, and its complete life cycle lasting eight days is well mastered in vitro. The main objective of our team is to understand better the global process of fruiting body development, named perithecia, induced normally in this species by fertilization. Three allelic mutants, named *pfd3*, *pfd9*, and *pfd23* (for “promoting fruiting body development”) obtained by UV mutagenesis, were selected in view of their abilities to promote barren perithecium development without fertilization. By complete genome sequencing of *pfd3* and *pfd9*, and mutant complementation, we identified point mutations in the *mcm1* gene as responsible for spontaneous perithecium development. MCM1 proteins are MADS box transcription factors that control diverse developmental processes in plants, metazoans, and fungi. We also identified using the same methods a mutation in the *VelC* gene as responsible for spontaneous perithecium development in the *vacua* mutant. The VelC protein belongs to the velvet family of regulators involved in the control of development and secondary metabolite production. A key role of MCM1 and VelC in coordinating the development of *P. anserina* perithecia with gamete formation and fertilization is highlighted.

## 1. Introduction

Ascomycota filamentous fungi differentiate complex multicellular fruiting bodies during sexual reproduction. Fruiting body formation usually requires a set of proper environmental conditions, such as light, temperature, and proper nutrients. In the ascohymenials, elaboration of most of the tissues of the fruiting body takes place after fertilization through precise developmental programs involving differentiated cells (for review [1,2]). On the contrary, in the ascoloculars, most tissues of the fruiting bodies are formed before the gametes, and hence before fertilization. The molecular mechanisms and the complete regulatory scheme leading to the production of mature fruiting bodies are not yet fully characterized despite numerous studies [2,3,4,5]. Different signal transduction pathways have been shown to control fruiting body development, and these include the MAP kinases, the STRIPAK complex, and the NADPH oxidases [1,5,6,7]. Recent comparative genomic and transcriptomic approaches have shown a large number of regulated genes during fruiting body development [8,9], but their roles remain to be determined. Although it produces fewer data than genomic methods, the classical approach of genetic analysis is a powerful tool to identify key genes involved in fruiting body development and, through a thorough phenotypic analysis, to obtain important information about their roles in the process.

The filamentous ascomycete *Podospora anserina* is a good model to study development of fruiting bodies (called perithecia in this species) through a direct genetic approach [2,7,10,11]. In *P. anserina*, the three-dimensional perithecia are issued from two types of cells. The envelope (or peridium) is derived from maternal vegetative haploid mycelium, whereas the dikaryotic zygotic tissue comes from both male and female parents and is embedded in the centrum (the tissues within the peridium that have both a maternal origin, such as the paraphyses, and a zygotic origin, i.e., the sexual lineage producing asci). Female gametangia, ascogonia, and male gametes, spermatia, are differentiated first (ascohymenial development). The ascogonium is rapidly surrounded by maternal hyphae and becomes a preformed fruiting body, the protoperithecium [2], which awaits fertilization. After fertilization, there is a coordinated growth of the maternal and zygotic tissues and perithecia begin to mature. After two days, necks and ostioles are formed and, after four days, maturation is completed with ascospore production and projection. There is thus a need to coordinate the developments of both the zygotic and maternal tissues in order to orchestrate the whole fruiting body development correctly.

In order to understand the developmental program leading to the development of perithecia in *P. anserina* better, mutageneses on wild-type homokaryotic strains, i.e., unable to engage in mating, have been performed to isolate mutants able to produce perithecia without prior fertilization. They have an identical phenotype to that of the long-known *vacua* mutant [12]. These mutants are called *pfd*, for “promoting fruiting body development”. We describe here the characterization of some of them that are in fact alleles of the same gene encoding the MADS box protein orthologous to the *Saccharomyces cerevisiae* MCM1 [13,14] and identify the gene affected in the *vacua* mutant as encoding the ortholog of *Aspergillus nidulans* VelC, a protein of the velvet family.

## 2. Materials and Methods

### 2.1. Strains and Growth Conditions

All strains of *P. anserina* used in this study are derived from the “S” (big S) wild-type strain that was used for sequencing of the *P. anserina* genome [15]. The most recent protocols for standard culture conditions, media, and genetic methods for *P. anserina* are described by Silar [16]. The Δ*mus51*::*phleoR* mutant strain differs from the S wild-type reference strain by a single deletion of the *mus-51* gene, which has increased frequency of targeted gene replacement [17]. The *pks1-193* strain has a mutation in the *PKS1* gene that encodes a polyketide synthase necessary for mycelium pigmentation [18]. The *Δmat* strain, lacking its mating type, is unable to engage in fertilization, but is fully able to provide hyphae for the building of the maternal tissues of the fruiting bodies [2,19]. These two last strains have been used in sexual crosses involving some *pfd* mutants and *mcm1^Δ^*. The *vacua* strain was purchased from ATCC (ATCC n° 42805). This mutant was obtained in the second strain of *P. anserina*: the s (small s) strain [12]. So, the first step was therefore to introgress the *vacua* mutation into the S (big S) background by ten successive crosses with the S strain as the female parent. In each successive generation, the 2:2 segregation of the *vacua* phenotype was observed, indicating as previously stated [12] that the phenotype was due to a single mutation.

### 2.2. Mutagenesis

Two UV mutageneses were made. In the first one (mutagenesis I), protoplasts (obtained as described in Silar [16]) of the S *mat-* strain were spread onto regeneration plates [16] at 10^4^/plate and irradiated with UV 256 nm at a dose of 250 J/m^2^, which resulted in the death of 99% of the protoplasts. The plates were then incubated at 27 °C in the dark. Thalli generated two days later by surviving protoplasts were transferred onto M2 plates at nine thalli per plate and incubated for a week at 27 °C in the presence of light (equivalent to daylight). Thalli generating perithecia/protoperithecia larger than those of the wild type were selected. Among 3000 tested protoplasts, 14 were found to contain a mutation enabling the development of large fruiting bodies, although some lack a neck and ostiole.

In the second round of mutagenesis (mutagenesis II), two-day-old S *mat+* thalli were fragmented in a Precess 24 (Bio-Rad, Hercules, CA, USA) (speed 4.0 for 20 s) and spread onto M2 medium. After an overnight incubation, the regenerating thalli were irradiated with UV 256 nm at a dose of 300 J/m^2^, protected from light overnight, and then incubated for a week at 27 °C in the presence of light. Large protoperithecia/fruiting bodies appearing on the plates were then transferred with some neighboring hyphae onto fresh M2 medium for further analysis. Thirteen additional mutants among 50 plates were recovered by this method. The resulting 27 mutants were then individually identified and numbered as *pfd* mutants, the term *pfd* meaning “Promoting Fruiting body Development” (Table 1).

All selected mutants were crossed to the wild type, and the *pfd* phenotype was shown to segregate as due to a single mutation. For some mutants, additional phenotypes co-segregated with the *pfd* phenotype, indicative of pleiotropy of the mutations. In this work, we will focus on the characterization of three allelic *pfd* mutants: *pfd3*, *pfd9*, and *pfd23* (see results).

### 2.3. Identification of Genes Mutated in pfd3, pfd9, and vacua Mutants

The Illumina sequencing work benefited from the facilities and expertise of the I2BC high throughput sequencing platform (formerly IMAGIF, https://www.france-genomique.org/plateformes-et-equipements/plateforme-de-sequencage-haut-debit-de-li2bc/, accessed on 4 December 2023). Custom-made libraries had 300 bp inserts and sequencing was 76-bp paired-end. Coverage was 85-fold. Sequence reads were mapped onto the reference genome of the S *mat+* strain [15] and potential mutations were detected using the galaxy server (https://usegalaxy.org, accessed on 4 December 2023). Briefly, fastq reads were mapped onto the S reference genome with Bowtie2 version 2.0.5 [20]. Polymorphisms with the reference sequence were identified with the samtools mpileup [21] and filtered with VarScan mpileup version 2.4.3 [22].

### 2.4. Characterization of the Mutation in pfd23 Mutants

To identify mutations in the *pfd23* mutants, the complete CDS of *mcm1* (*Pa_1_19280*) was amplified from mutant genomic DNA with primers mcm1-1F/mcm1-4R (Supporting Information Table S1), using a high-fidelity polymerase, and then sequenced.

### 2.5. Phylogenetic Analysis

Fungal genes orthologous and paralogous to *vacua* were searched for by BLAST 2.8.1 [23] in GenBanK and Mycocosm in February 2019 [24] using the default parameters, with the *vacua* protein as the query. Alignment was made with MAFFT V7.419 [25] and manually refined with Jalview 2.10.2 [26]. This alignment was used to construct a phylogenetic tree using the maximum likelihood method with 100 bootstrap replicates (PhyML software V2.4.4 using the default parameters) [27] and transferred to the iTOL server V5.1 for visualization [28].

### 2.6. Deletion of mcm1 and ste12 Genes

The deletion cassette of *mcm1* (*Pa_1_19280*) was constructed, as previously described [29]. The construction of the *ste12* (*Pa_7_1730*) deletion cassette was based on the *Asc*I/*Mlu*I method [30], modified as described by Coppin et al. [31]. A hygromycin resistance cassette was used for both. Protoplasts from the *mus51^Δ^::phleoR* and *mus51^Δ^::su8* were transformed by the deletion cassettes of *mcm1* and *ste12*, respectively. Transformants were selected on medium containing 75 μg/mL hygromycin. Putative deleted mutants were first selected by PCR. The PCR amplified specific junctions to the replaced locus, using two primers that annealed at one end of the hygromycin resistance gene and upstream of the proximal flank used in the deletion cassette (Supporting Information Table S1). Homologous recombination of the deletion cassette allowed amplification of a predictable fragment on each side of the selectable resistance gene. Two or three candidate transformants were genetically purified by crossing them to the wild-type strain to eliminate potential untransformed nuclei and to segregate out the *mus-51^Δ^* mutation. Several *mat+* and *mat-* strains containing the deletion but lacking *mus-51^Δ^* were selected from each progeny and subjected to Southern blot analysis for final validation (Supporting Information Figure S1). Purified transformants verified by Southern blot were chosen as stock deletion mutants for subsequent studies. For *mcm1*, transformation experiments were performed in both mating types. The double mutants *mcm1^Δ^ste12^Δ^*, in both mating types, were constructed by genetic crosses, and relevant genotypes were confirmed by PCR.

### 2.7. Complementation of the pfd3, pfd9, pfd23, mcm1^Δ^, and vacua Mutants

To ensure that the phenotypes observed for *mcm1* mutants were actually due to inactivation of the relevant gene, a complementation procedure was performed by ectopic insertion of the corresponding wild-type allele under control of its native promoter. A 1952 bp-DNA fragment carrying the wild-type *mcm1* CDS (*Pa_1_19280*) and its 5′ and 3′ flanking regions was amplified with primers mcm1-1F/mcm1-4R (Supporting Information Table S1), using a high-fidelity polymerase. The DNA fragment was cloned at the *Eco* RV site of the pBC-Phleo plasmid [32] to yield plasmid pBC-mcm1. Protoplasts of the *mcm1****^Δ^*** *mat+* mutant strain were transformed with this plasmid. Thirty-one transformants were selected on medium containing 10 μg/mL phleomycin. They were crossed to the *mcm1****^Δ^*** *mat-* strain. Eighteen transformants recovered a wild-type phenotype. Lack of fertility in the thirteen remaining transformants was likely due to either DNA rearrangements during integration and/or lack of expression at the insertion point. Three fertile transformants were selected and crossed with the wild type. Progeny was recovered and analyzed to identify F1 descendant strains carrying a *mcm1* wild-type transgene associated with the *mcm1* mutant allele or *mcm1* wild-type allele. These strains were then used to verify complementation for all *mcm1* mutants by crosses.

To complement the *vacua* mutation, the wild-type *Pa_7_6330* allele was amplified by PCR using primers vacuAfor and vacuArev (Supporting Information Table S1) on genomic DNA from the S strain. The *vacua* mutant was then co-transformed using 10 µg of the amplified product and 2 µg of pBC-hygro. Seventeen hygromycin B-resistant transformants were obtained, among which one complemented the *vacua* phenotype, i.e., did not spontaneously produce large perithecia.

### 2.8. Generation of the mcm1 Over-Expression Strains

To overexpress *mcm1*, three DNA fragments were amplified with Thermo Scientific Phusion High-Fidelity DNA Polymerase: a 750 bp DNA fragment carrying the promoter of the *AS4* gene using primers pBC-rev/AS4_spod1R, a 941 bp-DNA fragment carrying the CDS of *mcm1* with primers Surexmcm1-For/Surexmcm1-Rev, and a 216 pb-DNA fragment carrying the ribP2 terminator with primers TT_mcm1/pBC-For. All the primers are summarized in Table S1. These three fragments were fused by PCR with primers pBC-rev/pBC-For. The DNA fragment was cloned into the pBC-Geneticin plasmid [33], previously digested by *EcoRV* to yield pBC-surexmcm1. This plasmid was transformed into the protoplasts of the *mcm1****^Δ^*** *mat+* mutant strain. Twenty-one transformants were selected on medium containing 250 μg/mL geneticin. They were crossed to the *mcm1****^Δ^*** *mat-* mutant. Seven transformants recovered a wild-type phenotype. Progeny of three transformants crossed with the wild-type strain were recovered, and F1 descendants carrying an overexpressed *mcm1* transgene associated with the *mcm1* wild-type allele were selected for analysis. The resulting strain was named *surexmcm1.*

### 2.9. Construction of the mcm1-GFP Strains

To express the *mcm1-GFP* gene, three DNA fragments were amplified with Thermo Scientific Phusion High-Fidelity DNA Polymerase: a 741 bp DNA fragment carrying the promoter of the *AS4* gene using primers AS4-1F/AS4_mcm1R, a 896 bp-DNA fragment carrying the CDS of *mcm1* with primers Mcm1_AS4_F/Mcm1_fluo_R, and a 956 pb-DNA fragment carrying the CDS of GFP associated with the ribP2 terminator with primers Fluo_97F/TT_216R. All the primers are summarized in Table S1. These three fragments were fused by PCR with primers AS4-1F/TT_216R. The DNA fragment was cloned into the pBC-Geneticin plasmid [33], previously digested by *EcoRV* to yield the pBC-*mcm1_GFP* plasmid. This plasmid was transformed into the protoplasts of the *mcm1****^Δ^*** *mat+* mutant strain. Seven transformants were selected on medium containing 250 μg/mL geneticin. They were crossed to the *mcm1****^Δ^*** *mat-* mutant. Three transformants recovered a wild-type phenotype, demonstrating that the addition of eGFP did not modify the activity of *mcm1*. Progeny of these transformants crossed with the wild-type strain were recovered and a dozen F1 descendants carrying the *mcm1-GFP* transgene associated with the *mcm1* allele were selected for analysis. The strains were crossed with the wild type, and *mat+* and *mat-* strains expressing MCM1-GFP were selected in the progeny.

Strains expressing both MCM1-GFP and a histone H1-mCherry fusion protein were recovered in the progeny of a cross between a strain carrying MCM1-GFP and a strain carrying the histone H1-mCherry fusion protein previously constructed [34].

### 2.10. Growth and Development

To determine the role of MCM1 in fungal physiology, the mutated and deleted mutants and the wild-type strain were incubated separately on M2 medium at 27 °C. Colony morphology, pigmentation, perithecium formation, ascospore production, ascospore dispersal, and germination were observed during their vegetative growth and their sexual cycle.

Perithecium content was fixed in 7.4% paraformaldehyde and analyzed, as in Coppin et al. [31]. Microscopic observations with eGFP and mCherry were made on mycelia grown on agar. Pictures were taken with a Leica microscope (Bannockburn, IL, USA) DMIRE2; LED Lumencor (optoprim): eGFP (485/20) at the ImagoSeine Plateform of the Institut Jacques Monod (https://www.ijm.fr/technological-resources-and-facilities/platforms/imagoseine/?lang=en, accessed on 4 December 2023) or with a Nikon eclipse Ci microscope. Pictures were analyzed with ImageJ (Wayne Rasband, NIH, http://imagej.nih.gov/ij, accessed on 4 December 2023).

Microscopic observations of perithecia were made of mycelia grown on agar and images were captured with a home-made microscope, combining a camera (monochromatic 1/3” CMOS sensor with a resolution of 1280 × 960 pixels) and a telecentric objective (4× magnification and 65 mm focal length). The precision of the microscope was measured to be 1.10 micro-m/pixel. Enlightenment was achieved using the beam from a white LED light passing through the sample.

Perithecia enumeration and area estimation were performed using Aphelion Imaging Software V4.4.0 (ADCIS SA and Amerinex Applied Imaging), the different steps of which are detailed in Supporting Information Figure S2. Anastomoses were analyzed, as in Chan Ho Tong et al. 2014 and in Brun et al. 2009 [33,34].

### 2.11. Sensitivity to Various Stresses

Sensitivity of the mutated and deleted mutants was tested on sorbitol (0.55 M), KCl (0.25 M), NaCl (0.25 M), Congo red (0.7 mM), calcofluor (50 µM), and methylglyoxal (2.5 mM).

Sensitivity to antifungals was measured with Etest^®^ gradient strips (bioMérieux, Marcy l’Etoile, France) with fluconazole (0.016–256 µg/mL), amphotericin B, and itraconazole (0.002–32 µg/mL). The Etest^®^ assay was performed in accordance with the manufacturer’s instructions. Plates were inoculated with mycelia fragmented in a Precess 24 (BioRad, Hercules, CA, USA) onto the surface using glass beads, and the antifungal agent strips were placed on the plates. The Etest^®^ minimum inhibitory concentration (MIC) was recorded as the lowest concentration of an antifungal drug for which the elliptical zone of growth inhibition intersected the Etest strip. Microcolonies within the ellipse were ignored. The Etest^®^ MIC was recorded after incubation for 4 days.

## 3. Results

### 3.1. Mutant Recovery

“Promoting fruiting body development” (*pfd*) mutants were obtained via two UV mutagenesis rounds (and were selected for their ability to promote some perithecium development without fertilization). Twenty-seven mutants were selected. They were crossed to the wild-type strain and the descendants were analyzed. The mutant phenotypes with regard to fruiting body production and vegetative growth are summarized in Table 1. Where possible, the purified *pfd* mutants were crossed with themselves and, in all cases, they were crossed to the wild-type strain of the opposite mating type. The *pfd* mutants were affected at various stages of their vegetative and sexual development. Some strains had an altered mycelium, either in its aspect, as fluffy or flat, or in its color, as dark- or pink-pigmented, when compared with the wild-type strain (Table 1). Most *pfd* mutants produced only large neckless protoperithecia. Yet, few mutants produced additional normal-looking perithecia that turned out to be barren.

In three mutants, *pfd3*, *pfd9*, and *pfd23,* the mutations were shown to be closely linked to the mating type (d < 1 cM) and could not be dissociated from it. Therefore, *pfd3* and *pfd9* were only available as *mat-*, and *pfd23* as *mat+*. This observation led us to hypothesize that *pfd3*, *pfd9*, and *pfd23* could be affected in the same gene and they were chosen for further analyses. The *pfd9* and *pfd23* mutants presented many normal-looking and few large neckless barren perithecia. The third one, *pfd3*, presented a peculiar phenotype. Indeed, it was male- and female-sterile, yet it produced barren perithecia when present in heterokaryons with wild-type nuclei of the same mating type.

### 3.2. Mutations

**Figure 1:**
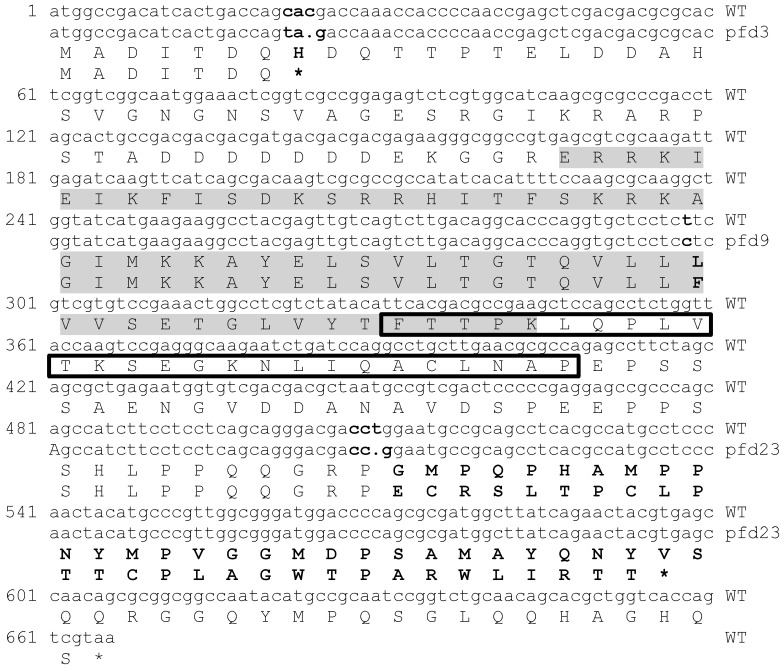
*Pfd* mutations in the *Pa_1_19280* gene and their outcome in the MCM1 protein sequence. The complete CDS of the *mcm1* wild-type (WT) strain is made up of 666 nucleotides (222 codons, 221 aa). Modifications in the genetic sequences of the three mutant strains and in the resulting proteins, when compared with the WT sequence, are shown in bold type. The deletion of a nucleotide is represented by a dot in the nucleotide sequence of the concerned mutants. The remarkable signature sequences for the MCM1 proteins, i.e., the MADS box and SAM domain, are marked in the figure by a shaded area and a boxed area, respectively. * are translation stops.

The *pfd3* and *pfd9* mutations were identified by direct genome sequencing. In each case, the mutant strain selected for sequencing originated from six generations of successive crosses between the mutant strain and the wild type. In this way, only 2.5% of the genome of the selected mutant strain came from the original mutant, then limiting the possibility of having mutations unrelated to the mutant phenotype. Point mutations were found for both mutants in the *Pa_1_19280* CDS, when compared with the wild-type strain. In *pfd3*, one nucleotide substitution (C to T, position 22) and one deletion (position 24) were found at the 8th codon, resulting in a stop codon at the beginning of the CDS (Figure 1). In *pfd9*, a T was mutated into a C at position 298, resulting in a substitution from a leucine to a phenylalanine at codon 99 (Figure 1). By sequencing the *Pa_1_19280* CDS in *pfd23*, one deleted nucleotide (position 510) was found, resulting in a frameshift at codon 170 (Figure 1). Overall, this confirmed that the three mutants were likely affected in the same gene.

Blast analysis revealed that *Pa_1_19280* likely encodes the *P. anserina* orthologue of the MCM1 protein, which displays identity values of 82%, 84%, and 80% with the *S. cerevisiae, Sordaria macrospora* and *Magnaporthe oryzae* proteins, respectively. Blast analysis of *P. anserina* MCM1 also allowed the localization of two highly conserved domains, the MADS box and the SAM domain, which are conserved in canonical MCM1 (Figure 1).

The *pfd9* mutation occurs in the highly conserved signature domain of the MADS box, whereas the *pfd23* mutation truncates the C-terminus, which could destabilize the protein. The *pfd3* mutation leads to a very short protein of 7aa and the next start codon that could be used to initiate translation is in the middle of the MADS box; *pfd3* is therefore likely a null allele.

### 3.3. Deletion of mcm1 and Complementation

In order to ascertain whether *pfd3* is a null allele and also to obtain a null allele of the opposite mating type, strains deleted in *mcm1* were constructed in both mating type by targeted gene deletion. Both deleted strains had indeed the same phenotype as *pfd3*, i.e., they were sterile and spontaneously produced barren perithecia, but only in heterokaryons with the wild-type nuclei of the same mating type.

To verify that the deletion of *mcm1* is responsible for the different phenotypes observed in the *mcm1^∆^* strain, a wild-type allele of *mcm1* was reintroduced in the deleted strains. The plasmid pBC-mcm1 carrying the wild-type *mcm1* gene as well as a phleomycin resistance cassette was thus transformed into the *mcm1^Δ^ mat+* strain. Among 31 phleomycin-resistant transformants, 18 had a wild-type phenotype: perithecia on the thalli of the complemented strains were recovered in crosses with *mcm1^Δ^ mat-* and no spontaneous perithecium was observed in heterokaryotic crosses with S *mat+*. However, among the 18 complemented transformants, 9 of them were partially complemented, as their perithecia were barren in heterokaryotic crosses with the *mcm1^∆^ mat-* strain. It is therefore possible that the location of the *mcm1* gene insertion into the genome may affect the expression of the transgene and lead to a total or partial complementation. Three fully complemented transformants were purified by crosses with the S *mat-* strain. In subsequent crosses, complemented strains *mcm1^Δ^* in both mating types were obtained. Crosses were also made with the different *pfd* strains, in order to verify if the wild-type allele was able to complement the different mutations. It did so for the three mutants, confirming that the mutations carried in *mcm1* by *pfd3*, *pfd9*, and *pfd23* were actually responsible for the *pfd* phenotype.

### 3.4. Sexual Development in the pfd Mutants and in mcm1^Δ^

**Figure 2:**
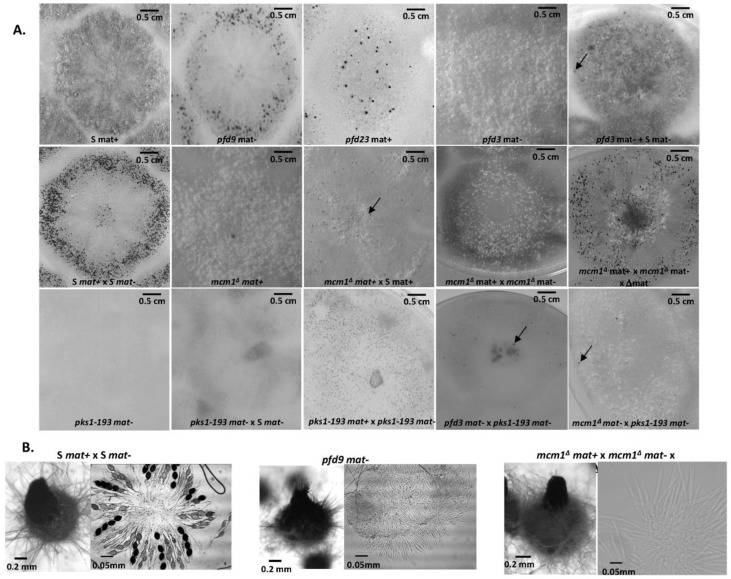
Spontaneous or fertilized perithecia (heterokaryotic crosses) in *pfd* mutants and *mcm1^Δ^*. (**A**). The pictures were taken 7 days after fertilization, at which time fully mature perithecia expel ascospores. Perithecia are visible as small dots in the close-up view. Arrow indicates *pfd3* and *mcm1^Δ^* spontaneous perithecia. (**B**). Microscopic observations of perithecia and typical rosette of asci observed in S fertilized perithecia and paraphyses observed in spontaneous perithecia after 7 days of growth in *pfd9* and in *mcm1^Δ^* crosses in the *^∆^mat* strain context. The *pfd9* mutant presents various sizes of spontaneous perithecia, some of which display sizes similar to wild-type fertilized perithecia, as shown here.

**Figure 3:**
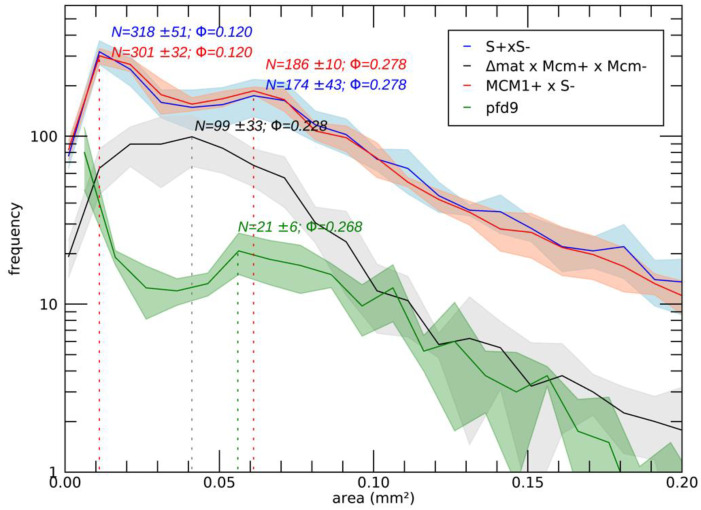
Number and variation in the size of fertilized perithecia after 10 days of growth (with N the average number of perithecia and Φ their equivalent diameter in mm). Perithecia metrics were extracted using Aphelion Imaging Software. For each strain, four independent replicates were performed. For details, see Supporting Information Figure S2.

In order to study the role of MCM1 in the development cycle of *P. anserina*, the production of perithecia, as well as their structure and their content, was carefully observed, as it is classically performed ([16]; Figure 2). Firstly, production of perithecia by the *pfd* mutants was tested on G medium (medium inducing ascospore germination, see [16]), in the dark and at 30 °C. In these three conditions, the wild type did not produce perithecia, nor did the *pfd* mutants, including *pfd3*, even when associated with wild-type hyphae of the same mating type. Then, in a more original way, for each experiment, the number and the size of perithecia were carefully measured with the Aphelion software, able to detect, count, and measure small objects, such as perithecia on a Petri dish, on four independent thalli after 7 and 10 days of culture. This kind of thallus cartography allowed us to quantify the production of perithecia in the wild-type strain and in various mutants in different contexts, and allowed us to complete the observations made microscopically usefully. The details of the procedure and all the relevant measurements performed are shown in Supporting Information Figure S2. Similar results were obtained after 7 and 10 days of culture, showing that smaller perithecia sizes could not be attributed to a sexual cycle longer than the time required to obtain perithecia in a wild-type cross (7 days). Figure 3 shows the number of perithecia (frequency) as a function of the measured area (in mm^2^) after 10 days of culture using for each experiment with the four thalli. Typically, in the wild-type cross, we can clearly see two peaks, corresponding to two populations of fruiting bodies: (i) protoperithecia that have not matured (average number = 318 ± 51, average area ~0.001 mm^2^) and (ii) mature perithecia (average number = 174 ± 43, average area ~0.06 mm^2^). Note that the average area values obtained in this case for perithecia from a wild-type cross are consistent with those measured microscopically (Figure 2B).

The *pfd9* and *pfd23* mutants were able to make spontaneous perithecia on their own (Figure 2A). As expected, since there was no fertilization, all spontaneous perithecia observed in mutant strains did not contain ascospores. Even if the size of these structures remains small, there is formation of true necks and tuft of hairs, which are characteristics of *P. anserina* perithecia [12]. These structures were filled with paraphyses (Figure 2B). The mycelium of *pfd9* and *pfd23* presented with the same pigmentation as the wild-type strain, with few aerial filaments for *pfd9* and a mycelium that appeared more aerial for *pfd23*. Neither of them presented with male or female sterility. In both cases, we counted a rather small number of perithecia (average number = 21 +/−6 for *pfd9*, Figure 3) but a rather wide distribution of surfaces around an average area of ~0.055 mm^2^ for *pfd9* (Figure 3), which showed that we had a population of spontaneous perithecia in a lower number and smaller size than mature perithecia but clearly bigger than the protoperithecia measured in a wild-type cross.

As previously stated, the *pfd3 m*utant was found to be peculiar. Indeed, in the homokaryotic progeny of a cross between *pfd3* and the wild type, we could not find any thalli forming perithecia, while the original mutant formed repetitively wild-type-looking perithecia. In fact, the *pfd3* mutant was only able to make spontaneous perithecia in heterokaryotic mycelium with wild-type nuclei of the same mating type (Figure 2A), and the original mutant was likely a heterokaryon carrying wild-type and mutant nuclei. The *pfd3* strain pigmented quickly and its mycelium had few airborne filaments compared with the wild-type strain. Similar phenotypes were observed for both *mcm1^Δ^* strains.

Spreading experiments involving *pfd3* x S or *mcm1^Δ^* x S were carried out and showed male and female sterility for the mutant strains. Indeed, no perithecium was observed on the mycelium of *pfd3* or *mcm1^Δ^* strains nor on the mycelium of the wild-type strain. No male (spermatium) or female (ascogonium) gametes were observed on the mutant thallus, probably indicating that these mutants were unable to differentiate these structures. Perithecia were only observed at the confrontation between the two strains. They were probably obtained by direct fertilization of ascogonia from the wild-type strain with mycelial filaments of the mutant strain. Another explanation was that heterokaryons may have been formed and complementation occurred where both strains met.

Then, the particularity of the *pfd3* mutant is that it needs wild-type nuclei of the same sexual type to make spontaneous perithecia. To understand better whether these perithecia originated from the mycelium of the mutant or that of the wild strain, a heterokaryon was constructed with *pfd3* and the *pks1-193* strain of the same mating type. This strain has a mutation in the *PKS1* gene that encodes a polyketide synthase necessary for mycelium pigmentation [18]. Perithecia from the mycelium of this strain are pink, while perithecia from a wild-type mycelium are dark green and pigmentation is cell-autonomous. We observed that *pfd3 mat-* X *pks1-193 mat-* heterokaryons were able to produce spontaneous barren perithecia that were fully pigmented dark green. Thus, we can conclude that these perithecia were made up solely of hyphae coming from the *pfd3* mutant, but were produced only if the mycelium contained a functional MCM1.

The *mcm1^Δ^* mutant did not produce spontaneous perithecia when cultured alone (Figure 2A). As with the *pfd3* mutant, the *mcm1^Δ^* nuclei must be in heterokaryons with wild-type ones of the same sexual type to allow spontaneous perithecia production. In heterokaryons with the *pks1-193* strain, the spontaneous perithecia observed were dark green, indicating that maternal tissue was provided by *mcm1^Δ^* hyphae and that wild-type nuclei were necessary for the formation of these unfertilized perithecia (Figure 2A). As with the three *pfd* mutants, the spontaneous perithecia from the *mcm1^Δ^* strain did not contain spores and were filled with paraphyses. In spreading crosses with a wild-type strain of the complementary mating type, they also displayed male and female sterility, with a very low production of gametes, spermatia, and ascogonia. Finally, *mcm1^Δ^ mat+* X *mcm1^Δ^ mat-* crosses were completely sterile. In order to understand better at which stage of sexual development the *mcm1^Δ^* strain is affected, we measured fertility in the *mcm1^Δ^ mat+* X *mcm1^Δ^ mat-* X *Δmat* trikaryon. This strain is unable to engage in fertilization, but is fully able to provide hyphae for the building of the maternal tissues of the fruiting bodies [2,35]. Here, a partial rescue in the trikaryon was observed, indicating that *mcm1* was required in the mycelium and/or the maternal tissues of the fruiting bodies for the formation of the perithecium envelope (Figure 2A). However, ascospore production was not improved in the trikaryon, showing that *mcm1^Δ^ mat+* X *mcm1^Δ^ mat-* zygotic tissue was also defective. Finally, we showed that heterokaryotic crosses *mcm1^Δ^ mat+* x S *mat-* (Figure 3) and *mcm1^Δ^ mat-* x S *mat+* behave as a wild-type cross in terms of number and size of protoperithecia and perithecia, meaning that, indeed, *mcm1^Δ^* shows normal sexual reproduction when crossed to the wild-type strain of the opposite mating type. Moreover, in the trikaryon *mcm1^Δ^ mat+* X *mcm1^Δ^ mat-* X *Δmat,* we observed a single peak, quite large (average number = 99 ± 33, average area ~0.04 mm^2^), corresponding to a partial rescue in the trikaryon, with a population of perithecia in a lower number and smaller size than in a wild-type cross (Figure 3).

### 3.5. Sensitivity to Various Stresses

Because the *mcm1* gene was previously shown to be involved in cell wall and osmotic stresses in *S. cerevisiae* [36], we evaluated resistance/sensitivity of the mutants to these various stresses as well as to oxidative and temperature stresses. Stress tests were carried out on the *pfd* and *mcm1^Δ^* mutant strains, the complemented strain and the *surexmcm1* strain, in which *mcm1* CDS is under the control of the strong and constitutive promoter of the *AS4* gene encoding the translation elongation factor EF1α. Each experiment was performed in triplicate. As the *surexmcm1* strain behaved throughout the experiments as the wild-type strain, this strain was not further analyzed. All tested strains behaved like the wild-type strain under osmotic (sorbitol, NaCl and KCl) and oxidative (methylglyoxal) stress conditions.

**Figure 4:**
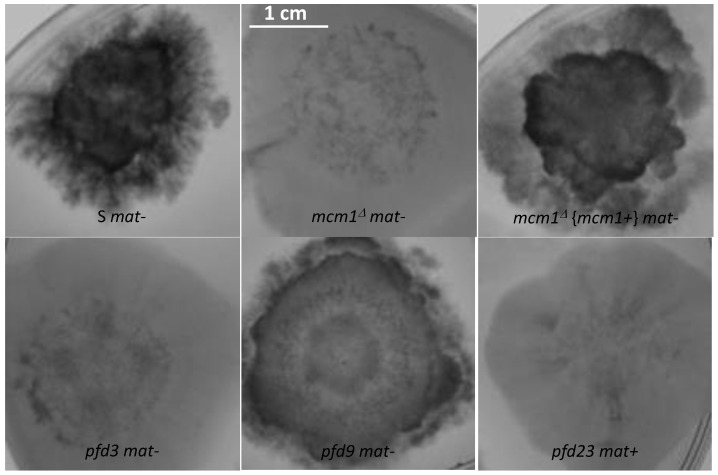
Effect of calcofluor (50 μM) on mycelial growth. Mutant and wild-type strains were observed after 3 days.

Calcofluor, which binds to chitin, and Congo red, which inhibits glucan synthases of the cell wall, had similar effects on the cell wall of the mutants (Figure 4). Briefly, the complemented strain behaved like the S wild-type strain, with a dark-pigmented mycelium and severely impaired growth. By contrast, the *pfd3*, *pfd23*, and *mcm1^Δ^* mutants showed normal growth that did not appear to be affected by the presence of both compounds. To a lesser extent, the *pfd9* mutant seemed to have an intermediate phenotype with little growth impact, only exhibiting a dark-pigmented mycelium.

**Figure 5:**
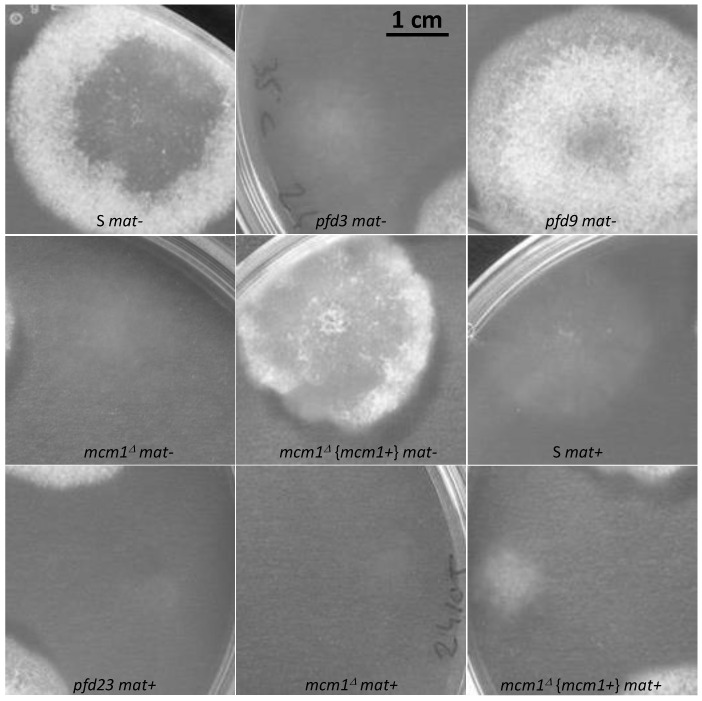
Effect of high temperature (35 °C) on mycelium growth. Pictures were taken after 3 days of growth.

Temperatures below (18 °C) and above (35 °C) the optimal growth temperature (27 °C) for *P. anserina* were tested. At 18 °C, all strains behaved in the same way as the wild-type strain. At 35 °C (Figure 5), the usual heat resistance of the S *mat-* strain was found when compared with the S *mat+* strain, as previously described [12]. This phenotype of heat resistance was found in *pfd9 mat-* but not in *pfd3* mat- nor in *mcm1^Δ^ mat-*.

### 3.6. Effects of Antifungals

As MCM1 was previously described to be involved in azole antifungal resistance in *Candida albicans* [37], resistance tests to two azole antifungals (fluconazole and itraconazole) and a polyene (amphotericin B) were evaluated with the different strains using the Etest strip technique.

**Figure 6:**
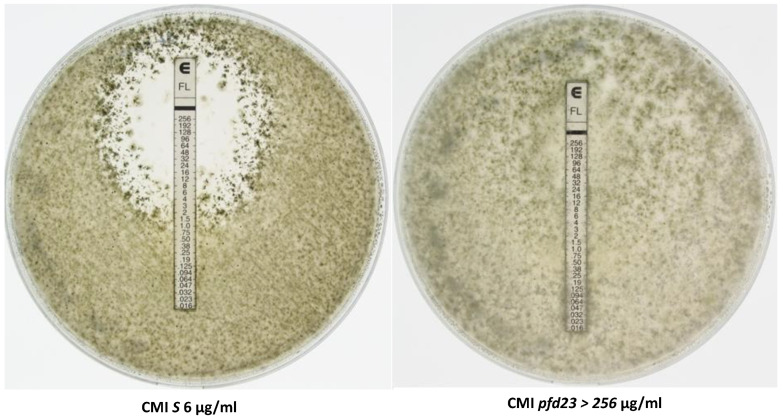
Fluconazole Etest. Each experiment was performed in triplicate. One hundred μL of fragmented mycelia from a 5 × 5 mm standardized plug was spread on the Petri dish. Pictures were taken after two days of growth.

Antifungal tests with fluconazole are shown in Figure 6 and in Table 2. The S strain displayed a MIC of 6 µg/µL. The *pfd3*, *pfd9*, and *mcm1^Δ^* mutants showed a moderate resistance, with a MIC of 12 µg/µL for *pfd3* and 16 µg/µL for *pfd9* and *mcm1^Δ^*, while the *pfd23* strain had a high resistance (MIC > 256 µg/µL). Tests with itraconazole yielded similar results (Table 2). Tests with amphotericin B showed that the *pfd3* and *mcm1^Δ^* mutant strains behaved as the wild-type strain. The *pfd9* and *pfd23* mutants had a moderate resistance to amphotericin B, with a MIC of 0.19 µg/µL and 0.25 µg/µL, respectively.

### 3.7. Localization of MCM1

**Figure 7:**
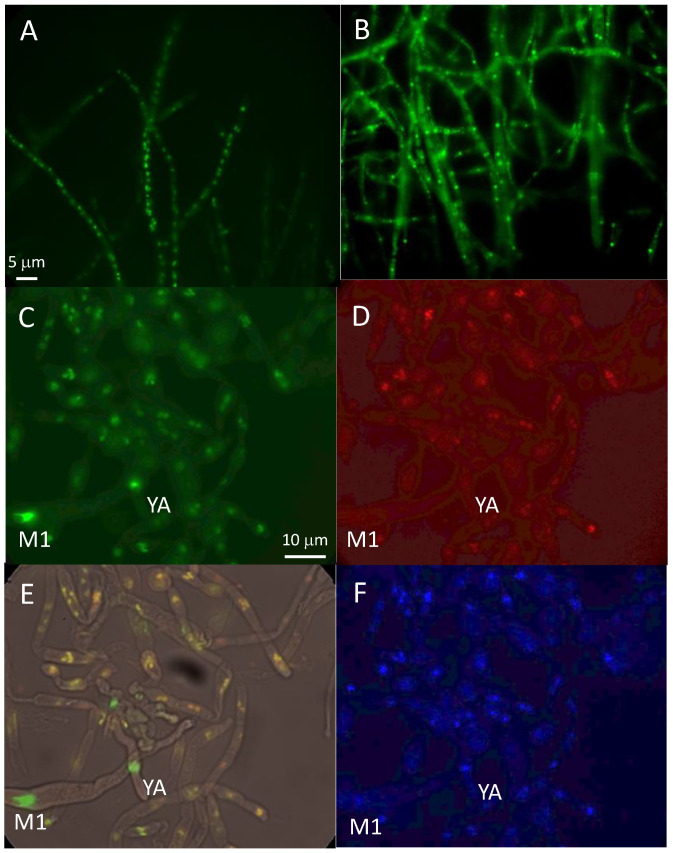
Localization of MCM1. A-B PaMCM1-GFP in vegetative phase of the *mcm1^∆^ mat+* strain, (**A**) exponential phase, (**B**) stationary phase. (**C**–**F**) PaMCM1-GFP during sexual development, C PaMCM1-GFP; D- H1-mCherry; E- merge; F DAPI. YA: young ascus, M1: ascus in prophase of meiosis 1.

After transformation of the *mcm1^∆^ mat+* strain to verify that the MCM1-GFP fusion protein was functional, we observed a punctuated marking, probably corresponding to nuclear localization, in the transformants that regained a wild-type phenotype (Figure 7A,B). To confirm this, a cross with the strain carrying the MCM1-GFP with a strain possessing the histone H1-mCherry fusion protein, previously constructed [34], was realized. Images taken by epifluorescence microscopy are shown in Figure 7C–F. Punctuated fluorescence was observed inside the perithecia. Co-location experiments with H1-mCherry and DAPI staining show that distribution of the MCM1 protein was compatible with a nuclear location.

### 3.8. Interaction with vacua

**Figure 8:**
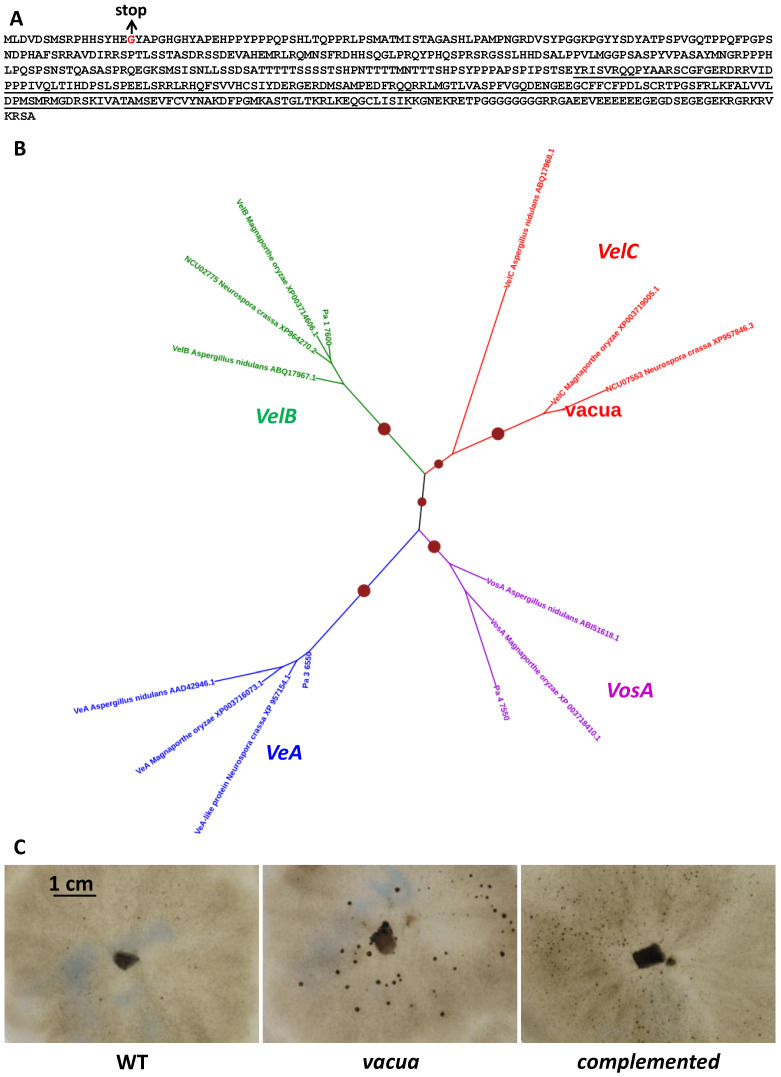
The *vacua* gene encodes a velvet protein. (**A**). protein sequence of the *Pa_7_6330* CDS. The velvet domain is underlined and the position of the mutation in the *vacua* mutant is indicated in red. (**B**). Phylogenetic tree of *vacua* and its homologues. The tree was constructed with PhyML using the WAG model. Dot sizes are proportional to bootstrap values. Proteins used were *P. anserina* VeA = Pa_3_6550, VelB = Pa_1_7600, VelC = *vacua* and VosA = Pa_4_7550*, Neurospora crassa* Ve1 protein (= VeA- like protein; GenBank accession n° XP_957154.1), Ve2 (=VelB, CDS number NCU02775), and a protein similar to *VelC (*CDS number NCU07553), *Magnaporthe grisea* VeA (GenBank accession n° XP_003716073.1), VelB (GenBank accession n° XP_003714606.1), VelC (GenBank accession n° XP_003719005.1) and VosA (GenBank accession n° XP_003718410.1) and *A. nidulans* VeA (GenBank accession n° AAD42946.1), VelB (GenBank accession n° ABQ17967.1), VelC (GenBank accession n° ABQ17968.1) and VosA (GenBank accession n° ABI51618.1), (**C**). Unlike the wild type (WT), the *vacua* mutant differentiated normal-looking barren perithecia without fertilization (mature-like sterile fruiting bodies). Introduction of a wild-type copy of the *vacua* gene prevented formation of mature-like perithecia (complemented strain); however, the complemented strain differentiated slightly larger neckless protoperithecia, suggesting that complementation was partial.

The previously described mutant *vacua* [12] presents a phenotype similar to *pfd* mutants, i.e., the production of normal-looking barren perithecia without fertilization. As for the *mcm1* mutants, *vacua* did not produce barren fruiting bodies on G medium, in the dark and at 30 °C. However, the *vacua* mutation is not linked to the *mat* locus, indicating that it is not allelic with the *MCM1* mutations. To identify the mutation responsible for the *vacua* phenotype, the genome of the mutant was sequenced. Beforehand, the *vacua* mutant was backcrossed ten times to the S strain, because it was obtained in the s strain [12] that contains numerous polymorphisms with respect to the S strain. Sequence analysis revealed a single mutation that could potentially account for the *vacua* phenotype: a G to T transversion changing codon 16 GGA coding for glycine into a TGA stop codon in the *Pa_7_6330* CDS (Figure 8A). This suggested that the mutation had created a null allele. To ascertain that this mutation was the one responsible for the phenotype, the *vacua* mutant was co-transformed with the wild-type allele of *Pa_7_6330* and a plasmid carrying a hygromycin B resistance gene. Among the 17 recovered hygromycin B-resistant transformants, one regained a wild-type phenotype, i.e., did not differentiate large perithecia in the absence of fertilization (Figure 8C). This transformant was crossed to the wild type. Analysis of the progeny indicated that restoration of a wild-type phenotype and hygromycin B resistance co-segregated, and that integration had occurred at a locus unlinked to *vacua*, showing that the mutation in *Pa_7_6330* was indeed responsible for the *vacua* phenotype. Nevertheless, complementation was not complete since the complemented F0 and F1 strains differentiated small neckless protoperithecia that were slightly larger than those produced by the wild type (Figure 8C). *Pa_7_6330* was thus renamed *vacua*. It encodes for a 55-kd protein that contain a velvet domain (Figure 8A). Phylogenetic analysis involving *vacua* and its homologues in *P. anserina* and related fungi (Figure 8B) revealed that *vacua* encoded a protein orthologous to the *VelC* genes of other fungi and that *P. anserina* contained a full complement of velvet genes (*VeA* = *Pa_3_6550*, *VelB* = *Pa_1_7600*, *VelC* = *vacua* and *VosA* = *Pa_4_7550*), as previously described [38].

The double mutant *mcm1^Δ^ vacua* was obtained by crosses. As the *vacua* allele did not have a selection marker, the *vacua* locus from candidate hygromycin-resistant spores (*mcm1^Δ^*) were sequenced, after amplification by PCR.

**Figure 9:**
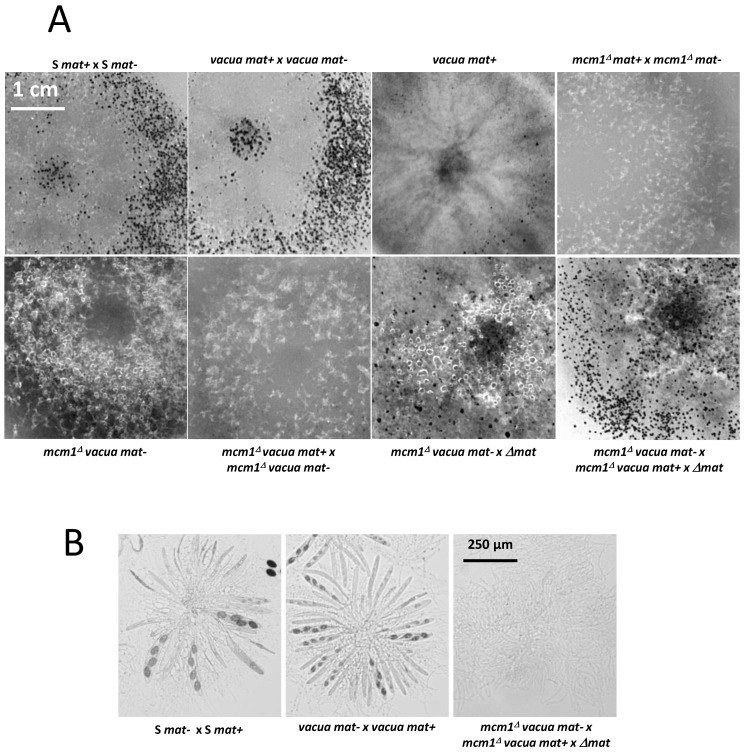
Heterokaryotic crosses involving the *vacua* and *mcm1^Δ^* mutants. (**A**). The pictures were taken 7 days after fertilization, at which time the fully mature perithecia expelled ascospores. Perithecia are visible as small dots in the close-up view. (**B**). Microscopic observations of perithecia and typical rosette of asci observed in S and *vacua* fertilized perithecia and paraphyses observed in the double mutant after 7 days of growth.

The double mutant *mcm1^Δ^vacua* spontaneously differentiated perithecia, although in lower amounts than the *vacua* mutant when alone, indicating that the mutation in *vacua* was epistatic over the null mutations in *mcm1* (Figure 9). Conversely, it produced almost no spermatia like the single *mcm1^Δ^* mutant.

### 3.9. Interaction with ste12

**Figure 10:**
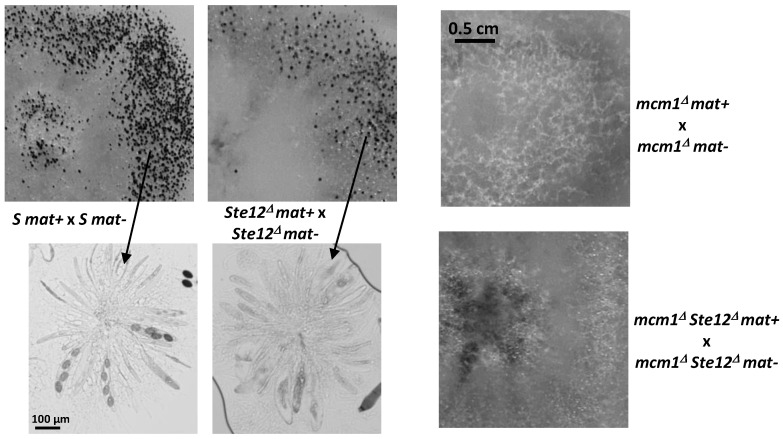
Heterokaryotic crosses involving the *ste12^Δ^* and *mcm1^Δ^* mutants. The pictures were taken 7 days after seeding, at which time fully mature perithecia expelled ascospores. Perithecia are visible as small dots in the close-up view. Below pointed by arrows microscopic observations of perithecia and typical rosette of asci observed in S perithecia and in *Ste12^∆^* perithecia, showing a small delay in sexual development.

As in *S. macrospora*, MCM1 has been shown to interact with the homeodomain transcription factor (HD) Ste12 during ascospore formation [39], and therefore we analyzed the mcm1/ste12 interaction in *P. anserina*. To this end, a *ste12^∆^* strain was constructed. This mutant presented a small delay in sexual reproduction (Figure 10 at day 7). It also featured a germination defect, as its germination rate was 58% instead of the 97% typical for the wild type.

The double mutant was obtained in the progeny of *mcm1^Δ^ x ste12^∆^* crosses. It presented the same phenotype as the single *mcm1^Δ^* mutant alone, i.e., the double mutant was sterile, indicating that mutations in *mcm1* were epistatic over mutations in *ste12*.

## 4. Discussion

Like in other fungi, perithecium differentiation in *P. anserina* is triggered by a proper set of environmental cues, e.g., light, temperature, adequate nutrients, etc., and proceeds mostly after fertilization, as seen in typical ascohymenial development. Indeed, the female gametangia stop their development as small protoperithecia and await fertilization. Upon fertilization, maturation of the fruiting bodies resumes and several tissues/specialized hyphae of maternal origin are then differentiated, including the neck, the paraphyses, the layers of the peridium, etc. This results in the mature perithecium that is several orders of magnitude larger than the protoperithecium. How development is coordinated to produce mature fruiting bodies properly ejecting ascospores is not fully understood. It was known from the recovery of the *vacua* mutant that maternal tissues can alone produce normal-looking, yet barren, perithecia, suggesting that the genetic maternal program(s) used to construct the fruiting bodies can be activated in the absence of fertilization. This advocates that ascohymenial and ascolocular developmental programs are not radically different and only differ in the timing of activation of these autonomous program(s) managing the development of the maternal tissue of the fruiting body. With the aim to understand how these program(s) are activated, we selected additional mutants with a phenotype similar to *vacua*. Identification of the genes affected in *vacua* and these newly isolated mutants should then help us to understand how development is initiated at the molecular level. To the best of our knowledge, only one other mutant from *Magnaporthe grisea* displays a similar phenotype, and the affected gene in this mutant is still unknown [40]. Note that such mutants can only be easily recovered in heterothallics or pseudohomothallics like *P. anserina* and *M. grisea* and not in homothallics such as *S. macrospora* and *A. nidulans*, in which many studies aimed at understanding the development of fruiting bodies are performed (see below).

Here, the identification of the genes affected in the newly-isolated *pfd3*, *pfd9*, *and pfd23* mutants and of the *P. anserina vacua* mutant showed the involvement of the *mcm1* and *VelC* genes in activating the development of perithecia. In these mutants, the ability to produce spontaneous perithecia is due solely to a bypass of fertilization, because they do not produce perithecia if the proper conditions are not met, i.e., the mutants still require availability of proper nutrients, an adequate temperature lower than 30 °C, and the presence of light to produce fruiting bodies.

The yeast MCM1 protein and its orthologues [14] belong to the MADS box protein family, with four other founding members, i.e., Arg80 (in yeast) [41], Agamous (in plants) [42], Deficiens (in plants) [43] and SRF (in humans) [44]; all contain a conserved DNA binding and dimerization domain named the MADS box. These proteins, described as cellular coordinators, are transcription factors present in most eukaryotic organisms. They control different developmental processes in plants, fungi, and animals. In animal and fungi, there are two distinct types of MADS box proteins, the SRF-like (type I) and MEF2-like (type II) classes [45]. MCM1 belongs to the SRF-like class. In *S. cerevisiae*, it was demonstrated to be involved in cell cycle control. This transcriptional factor is activated by the Bck2 protein and then induces expression of G_1_/S genes [46]. In this work, we showed that the MCM1 protein fused with GFP is localized in the nuclei of *P. anserina*. This is consistent with the prediction that the MCM1 protein is a transcription factor. In *S. macrospora*, as shown here in *P. anserina*, MCM1 controls different development processes and is essential for the formation of perithecia [47,48]. A similar role has been evidenced in *A. nidulans* [49], *Fusarium graminearum* [50], and *Colletotrichun fructicola* [51]. In the phytopathogen *M. oryzae*, the gene *MoMCM1* was shown to be required for appressorial penetration and male fertility, but it does not seem to be involved in fruiting body formation [52]. The role of MCM1 has also been investigated by gene deletion in *Beauveria bassiana* [53] and *Verticillium dahlia*, [54], but its involvement in fruiting body formation has not been assayed.

Interestingly, phenotypes are different in the *pfd* point mutants and in the *mcm1^Δ^* deleted strain. The *pfd9 mat-* mutant, which has a mutation in the sequence encoding the MADS box domain, can form perithecia spontaneously, i.e., without fertilization. The same phenotype is observed in the *pfd23 mat+* mutant, which has a shift in the open reading frame at the end of the coding sequence. On the other hand, the null *pfd3 mat-* mutant, which has a stop codon at the 8th codon, does not make spontaneous perithecia on its own. To observe such a phenotype, this mutant must be in heterokaryons with the wild-type strain. The same observations are made in *mcm1^Δ^*, where the *mcm1* gene has been deleted. Of importance, the null mutants (*pfd3* and *mcm1^Δ^*) are male and female sterile, without production of male or female gametes, whereas the point mutations in the *pfd9* and *pfd23* mutants do not appear to have strong effect on fertility. To account for this bizarre behavior, it can be hypothesized that the mutations present in the *mcm1* gene in the *pfd9* and *pfd23* mutants result in partially functional MCM1 proteins. These would fail to repress properly the commitment of protoperithecia to continue their development, hence the spontaneous appearance of barren perithecia; however, they would be functional enough to allow gamete production and the subsequent steps of the maturation of the fruiting bodies. The MCM1 would thus have two functions: the first one as a repressor of the maternal program(s) enabling the making of the maternal tissues of the fruiting bodies while the maternal gametes wait for fertilizing male gametes, and the second one as an activator permitting fruiting body formation. What programs are repressed and activated is not yet clear. MCM1 is required in *P. anserina* for the production of spermatia and ascogonia. Failure to produce perithecia in the null mutants when no wild-type *mcm1* nucleus is present could thus result solely from lack of gamete production. However, in *S. macrospora*, it has been shown that MCM1 is able to form homodimers and to interact with the mating-type protein SMTA-1, and then may be involved in the transcriptional regulation of mating-type-specific genes [47]; hence, MCM1 is likely required for “fertilization” in this homothallic fungus. This role may be conserved in *P. anserina*. It can be hypothesized that when wild-type nuclei are present, enough MCM1 protein would be supplied and diffuse into mutant hyphae to enable gamete production and/or fertilization. Once this critical step controlled by MCM1 is completed, perithecium maturation would continue because MCM1, whose gene is missing in such gametes, could not repress the development of maternal tissue.

As stated above, it has previously been shown that MCM1 interacts with the HD protein STE12, a transcription factor that activates genes of the mating pathway in yeast [39]. STE12 acts downstream of the MAP kinase FUS3/KSS1 cascade, where it plays an important role in mating and filamentation [55]. In *A. nidulans*, *Neurospora crassa*, and *S. macrospora*, STE12 also controls sexual development [39,56,57]. In *S. macrospora*, deletion of the *ste12* genes induces a defect in ascospore formation, while in *N. crassa* and *A. nidulans* the mutants fail to produce protoperithecia/cleistothecia. In *M. grisea*, the *STE12* mutants deleted for the *Mst12* gene are both male and female fertile [58]; however, homozygous crosses of the *STE12* mutants were not performed, preventing us from knowing whether STE12 is required for perithecium development, especially in the knowledge that, as in *S. macrospora*, MoMCM1 interacts with both Mst12 and Mata-1 [52]. In *P. anserina*, we show that the STE12 ortholog has a small role during sexual reproduction, since homozygous crosses of the mutant were fertile and only slightly delayed when compared with the wild type, the major defect being an impairment in ascospore germination. As would be expected from their phenotypes, the double mutant *mcm1^Δ^ ste12^∆^*presented the same phenotype as the single *mcm1^Δ^* mutant alone, suggesting that there is no interaction between Ste12 and MCM1 during the developmental process in *P. anserina*.

The role of the *VelC* gene has been investigated in *A. nidulans*, *M. grisea*, and *Valsa mali* [59], but its role in fertility was only assayed in *A. nidulans*, where it was shown that its deletion results in diminished production of cleistothecia, while its overexpression has the opposite effect [60]). In *N. crassa*, the *VelC* orthologue “*VE-3*” is described as being non-functional [61] and its deletion appears to have no effect (see the unpublished data mentioned in [38]). However, upon examination of the genome sequences of strain OR74, it appears that *VE-3* is badly annotated (see Supporting Information Figure S3), because an additional base is present within the coding sequence, creating a frameshift. This triggers the annotation to predict a false intron and the lack of part of the velvet domain in the predicted *VE-3* protein. The supplementary base is not present in the genome sequence of strain ORS-SL6a; hence, in this *N. crassa* strain, VE-3 appears fully functional and its role remains to be assessed. Here, we show that it is involved in *P. anserina* in controlling fruiting body development, as in *A. nidulans*. Based on the phenotype of the *mcm1^Δ^vacua* double mutant, it is likely that MCM1 and the vacua proteins operate either in independent pathways to bypass fertilization or more likely that vacua acts downstream of MCM1 in the same signaling pathway. Unlike the other members of the velvet family [61], there is at present no evidence to suggest that *vacua/Velc* is involved in light regulation as the *vacua* mutant did not produce mature fruiting bodies in the dark.

Phenotypic characterization continued by testing different types of stress on the mutants in this study. In particular, a previous study on the yeast *S. cerevisiae* showed that MCM1 is required for transcription of a subset of genes involved in maintenance of the cell wall and then is involved in resistance to calcofluor, a compound that binds to chitin in fungal cells [36]. The deleted strains, as well as the *pfd3* and *pfd23* mutants, appeared to be more resistant to calcofluor than the wild-type strain, which is the reverse of the phenotypes observed in yeast. The *pfd9* mutant showed a phenotype close to that of the wild-type strain, which would confirm that the MCM1 protein is partially functional in this strain. Tests with Congo red, a glucan synthase inhibitor, showed similar results. Further experiments must be undertaken to understand how MCM1 is involved in the regulation of fungal wall formation. Moreover, contrary to what is observed in *S. cerevisiae* [62], the MCM1 protein does not seem to be involved in osmotic stress resistance in *P. anserina*. Finally, none of the mutants tested showed any particular phenotype with respect to oxidative stress or growth at low temperature (18 °C). At 35 °C, it has been described that the S *mat-* strain is thermoresistant [63]. The *pfd3 mat-* and *mcm1^Δ^ mat-* mutants tested in this study both became thermosensitive at 35 °C. Thus, it appears that the MCM1 transcription factor directly or indirectly regulates the expression of the *RMP* gene, which is responsible for the 35 °C thermoresistance of the S *mat-* strain. It is interesting to note that the *RMP* gene is, like the *mcm1* gene, linked to the mat locus.

Antifungal testing on the different strains showed an increase in resistance to azole antifungals in link with deletion or mutations in the *mcm1* gene. These results are contradictory to the results obtained in *C. albicans* [64], in which deletion of *mcm1* caused hypersensitivity of strains to azoles. In *C. albicans*, it has been shown that azoles can be expelled normally from the cell through activation of MDR1 efflux pumps via the MCM1 transcription factor [64]. Then, it has been hypothesized that, when *mcm1* is deleted, pumps are no longer activated, and the antifungals are no longer properly expelled, which results in hypersensitivity to azole antifungals. In this study, *P. anserina* mutants appear to be more resistant to azole antifungals and to a lesser extent to polyene antifungals (amphotericin B). It is conceivable that, in this case, the transcription factor represses efflux pump expression in the S strain and that inactivation of *mcm1* causes deregulation of efflux pump expression or constitutive expression, resulting in higher resistance to antifungal drugs.

## 5. Conclusions

In conclusion, classical genetic analysis has permitted us to identify the MCM1 and VelC proteins as crucial for repressing maternal tissue development in *P. anserina*. According to the results provided by this study, the *mcm1* gene in *P. anserina* probably encodes a transcription factor, as suggested by its nuclear localization. In addition to repressing maternal tissue development, it also controls the production of both male and female gametes. Analysis of the interactions between the *mcm1* and *vacua* mutants tentatively suggests that *mcm1* and *vacua* act in the same pathway, with *vacua* acting downstream of *mcm1*.

## Figures and Tables

**Table 1:** The *pfd* mutants.

	Mutant	Fruiting Body Formation	Linkage	Other Phenotypes
Mutagenesis I	*pfd1*	Large neckless protoperithecia		Fluffy
	*pfd3 mat-*	Normal-looking perithecia when in heterokaryon with the wild type of same mating type	mat (d < 1 cM)	Flat and dark mycelium, male sterility, female sterility but small protoperithecia formed
	*pfd4*	Large neckless protoperithecia		Spindly mycelium that becomes wild type, homozygous crosses produce empty perithecia filled with jelly
	*pfd6*	Large neckless protoperithecia		Fluffy
	*pfd7*	Large neckless protoperithecia		Fluffy
	*pfd8*	Large neckless protoperithecia		None
	*pfd9 mat-*	Normal-looking perithecia and large neckless protoperithecia	mat (d < 1 cM)	None
	*pfd10*	Large protoperithecia some with neck initials		Slow and fluffy growth, homozygous crosses are partially sterile
	*pfd11*	Large neckless protoperithecia		Pink mycelium, homozygous crosses are sterile
	*pfd12*	Large neckless protoperithecia		Pink mycelium, homozygous crosses are sterile
	*pfd13*	Few normal-looking perithecia and large neckless protoperithecia		Homozygous crosses are sterile
	*pfd15*	Large neckless protoperithecia		None
	*pfd16*	Large neckless protoperithecia		Pink mycelium, homozygous crosses are partially sterile
	*pfd17*	Few normal-looking perithecia and large neckless protoperithecia		Fluffy, perithecia with abnormally shaped neck in homozygous crosses
Mutagenesis II	*pdf18*	Large neckless protoperithecia		None
	*pfd19*	Few normal-looking perithecia and large neckless protoperithecia		Fluffy, perithecia with abnormally shaped neck in homozygous crosses
	*pfd20*	Large neckless protoperithecia		Fluffy
	*pfd21*	Large neckless protoperithecia		None
	*pfd22*	Large neckless protoperithecia		None
	*pfd23 mat+*	Normal-looking perithecia and large neckless protoperithecia	mat (d < 1 cM)	Dark and fluffy mycelium
	*pfd24*	Large neckless protoperithecia		None
	*pfd25*	Large neckless protoperithecia		None
	*pfd26*	Large neckless protoperithecia		None
	*pfd28*	Large neckless protoperithecia		Pink mycelium
	*pfd29*	Large neckless protoperithecia		None
	*pfd30*	Small perithecia with large necks		Slow-growing mycelium
	*pfd31*	Few normal-looking perithecia and large neckless protoperithecia		None

**Table 2:** Antifungal Etests.

Strains	MIC Fluconazole	MIC Itraconazole	MIC Amphotericin B
*S*	6 µg/µL	0.5 µg/µL	0.047 µg/µL
*mcm1^∆^*	16 µg/µL	2 µg/µL	0.032 µg/µL
*pfd3*	12 µg/µL	1.5 µg/µL	0.032 µg/µL
*pfd9*	16 µg/µL	1.0 µg/µL	0.19 µg/µL
*pfd23*	>256 µg/µL	>32 µg/µL	0.25 µg/µL

## Data Availability

Data are contained within the article and supplementary materials.

## Supplementary Materials

The following are available online at https://www.mdpi.com/article/10.3390/jof10010079/s1, Supporting Information Figure S1. Southern blot analysis for final validation. Supporting Information Figure S2. Methodological approach for perithecia enumeration and area estimation made by Aphelion Imaging Software. Supporting Information Figure S3. Sequences of the *VelC* homologues of *N. crassa* in strains ORS-SL6a and OR74A. The additional base in OR74A is boxed in white. Bottom, domain analysis using CD-search (https://www.ncbi.nlm.nih.gov/Structure/cdd/wrpsb.cgi, accessed on 4 December 2023) of the *VelC* homologue of ORS-SL6a showing that in this strain the velvet domain is complete. Supporting Information Table S1. Primers used for *mcm1* (*Pa_1_19280*), *ste12* (*Pa-7-1730*) and *vacua* (*Pa-7-6330*) gene deletion, complementation, and detection. Supporting Information Table S2: Summarize of global measurements, based on perithecia surfaces (mm^2^); Count: total number of extracted objects for each replicate; area_mean and area_med are the mean value and median value of perithecia for each replicate; areas; std is the standard deviation of area datasets.
