# Supplementary material for: Mutations in Podospora anserina MCM1 and VelC Trigger Spontaneous Development of Barren Fruiting Bodies
Source: J Fungi (Basel). 2024 Jan 19;10(1):79. doi: 10.3390/jof10010079 (PMC10819945; doi:10.3390/jof10010079)

Figure S1. **Validation of deletion mutants.**

(A) Genomic DNA was isolated from wild type (WT) and the two mating type deletion transformants, and was then digested with the indicated restriction enzyme *Kpn*I. The blots were probed either with a sequence containing the relevant flanking region. A restriction map of the WT and mutant locus is presented. (B) The probes corresponding to the flanking regions are indicated in green on the maps. The sizes of the expected fragments are indicated on the maps and are reported close to the corresponding fragment on the southern blot.

A

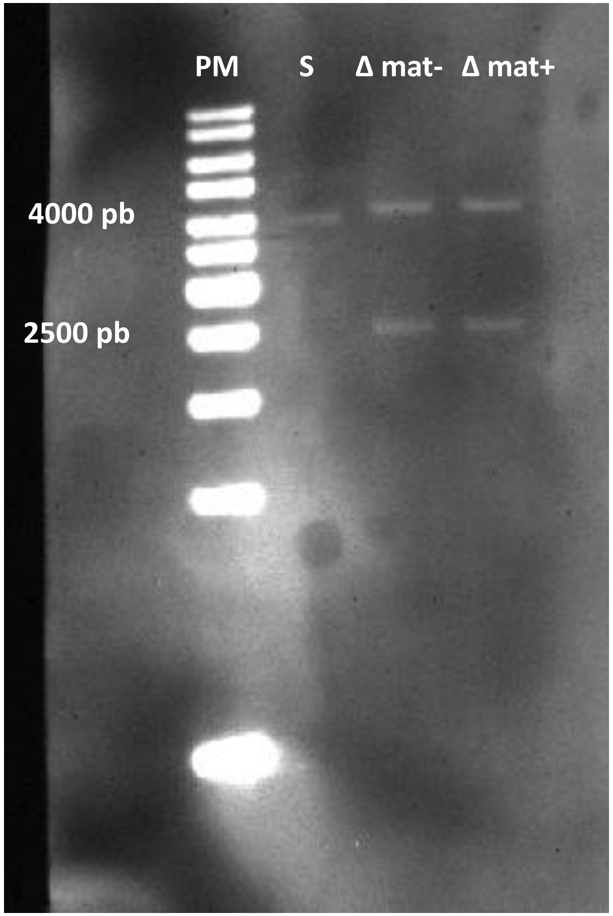

B

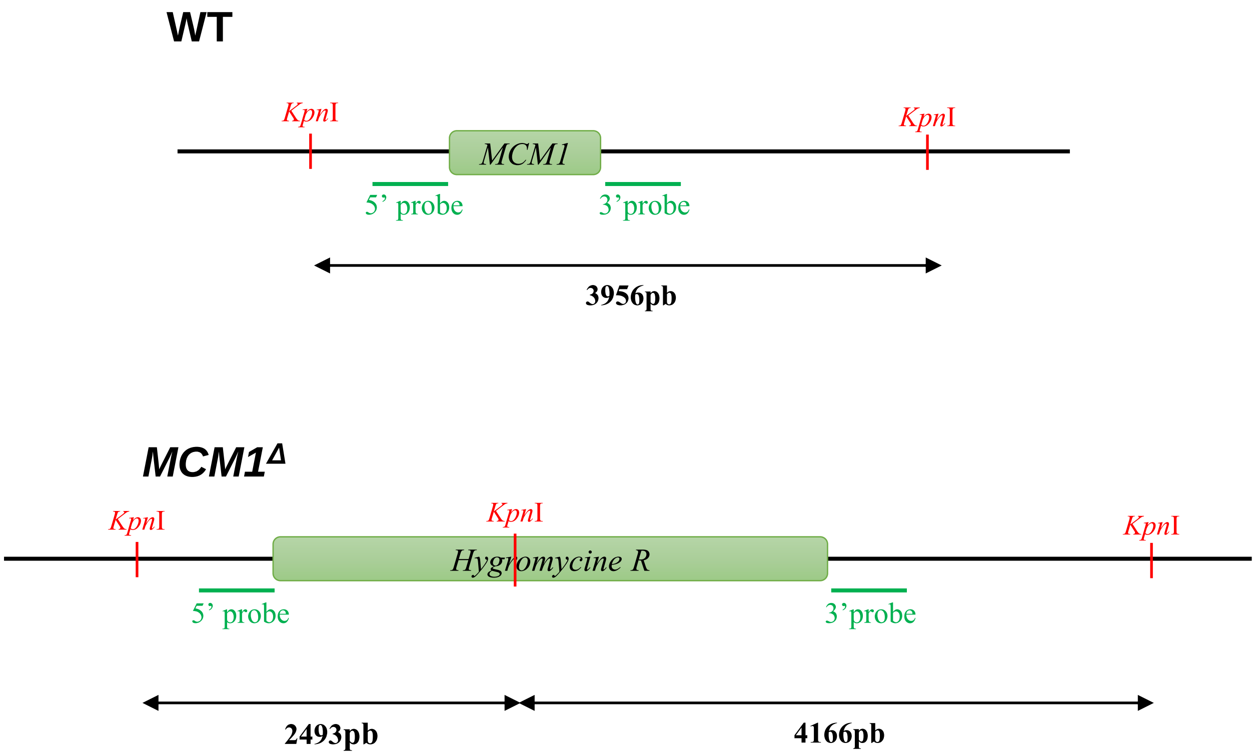

Supplement: Supplementary file 1 [file jof-10-00079-s001.zip › jof-2783895-supplementary1/Supporting Information Fig S1.pdf]
