# Supplementary material for: Mutations in Podospora anserina MCM1 and VelC Trigger Spontaneous Development of Barren Fruiting Bodies
Source: J Fungi (Basel). 2024 Jan 19;10(1):79. doi: 10.3390/jof10010079 (PMC10819945; doi:10.3390/jof10010079)
Supplement: Supplementary file 1 [file jof-10-00079-s001.zip › jof-2783895-supplementary1/Supporting Information Fig. S2 and Table S2.pdf]

Supporting Information Fig. S2.

Methodological approach for perithecia enumeration and area estimation made by Aphelion Imaging Software (ADCIS SA and Amerinex Applied Imaging),

The RGB images (Fig. S2A) are in a first step calibrated to convert measurements computed in pixel units to real world units; in our case, one side pixel corresponds to 0,03284 mm. For each image, the successive steps of image treatments may be described as follows:

i) We perform a threshold segmentation on the red channel of images (i.e. a grayscale image coded in 256 levels, black corresponding to the value 0 and white to the value 255), this latter having a good contrast between the dark perithecia objects and the brighter medium.
The defined threshold value, slightly varying from one image to another, is chosen in such a way that the perithecia detected do not form too large aggregated areas, as it can be observed in Fig. S2B. The brightest perithecia objects, having an intensity level too close to that of the medium, cannot be taken into account in this work (see details in fig. 2C).
A binary image is then computed: pixel values are equal to 1 for the darkest objects – the detected perithecia- when the pixel values for brightest areas are equal to 0 (the medium). Each value area equal to 1 on the binary image is then converted into an vectorised objet, on which statistics may be performed.

ii) Connected objects, as it may be observed in Fig. S2B, are automatically separated by using a watershed operator. This one consists in splitting connected objects into convex ones. We have to precise that many of large objects (agglomerated peritecia with a less elongated shape) could not be splitted by this operator, that can be observed on fig 2c. This explains the histogram tail (Fig 2d).

iii) Statistical measurements based on object areas, given in mm², are then computed (see the normalized histogram in Fig. S2D showing the distribution of perithecia areas). For each experiment, we performed statistical analyses on each thallii (except for the central one) separatly. The Mean, median and standard deviation of areas are given in Table S2.

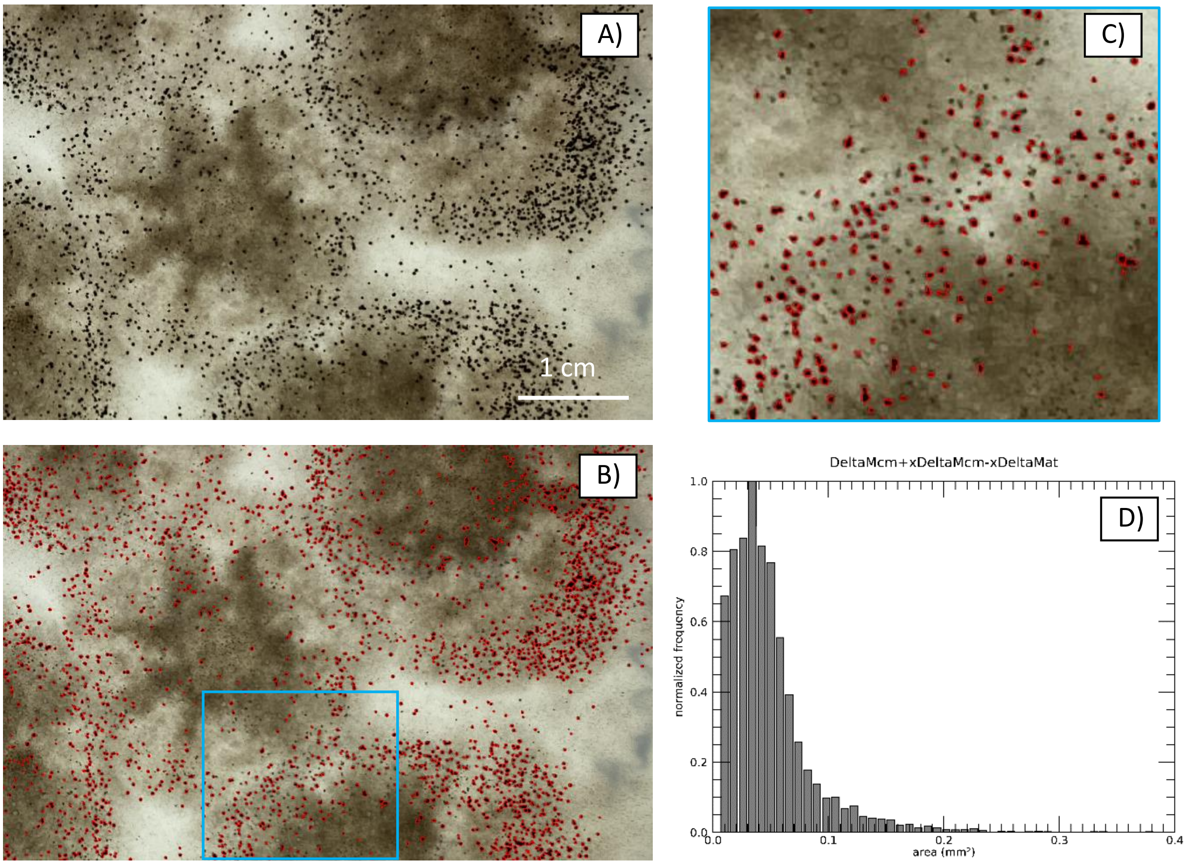

Figure S2: A) Portion of a RGB image showing isolated and agglutinated perithecia; B) Detection and extraction of dark objects by applying a segmentation by threshold; C) Zoom highligting the detected perithetia, located in the blue frame in B); D) Normalized histogram (for the tricaryon *mcm1*$^{\Delta}$ mat+ X *mcm1*$^{\Delta}$ mat- X *Δmat,* as example*)* representing the distribution of perithecia areas (binsize of 0.008).

Table S2: Summarize of global measurements, based on perithecia surfaces (mm²); Count: total number of extracted objects for each replicate; area_mean and area_med are the mean value and median value of perithecia for each replicate; areas; std is the standard deviation of area datasets.

Crosses	Strains involved	Replicate	count	area_mean	area_med	std
opposite mating types D7	*mcm1Δ+ x S-*	1	2510	0,0400	0,0349	0,0287
		2	2258	0,0396	0,0349	0,0279
		3	1962	0,0454	0,0383	0,0365
		4	2451	0,0394	0,0338	0,0275
opposite mating types D7	*mcm1Δ- x S+*	1	2367	0,0494	0,0372	0,0457
		2	1852	0,0485	0,0349	0,0499
		3	1759	0,0537	0,0406	0,0507
		4	2578	0,0451	0,0360	0,0373
opposite mating types D7	*S+ x S-*	1	2609	0,0782	0,0574	0,0816
		2	2111	0,0788	0,0574	0,0849
		3	1665	0,0770	0,0529	0,0835
		4	1241	0,0733	0,0541	0,0705
opposite mating types D10	*mcm1Δ+ x S-*	1	2309	0,0725	0,0529	0,0726
		2	2127	0,0740	0,0574	0,0730
		3	1934	0,0738	0,0563	0,0747
		4	2238	0,0730	0,0608	0,0670
opposite mating types D10	*mcm1Δ- x S+*	1	2141	0,0466	0,0372	0,0425
		2	1817	0,0478	0,0349	0,0512
		3	1744	0,0518	0,0372	0,0543
		4	2389	0,0374	0,0315	0,0320
opposite mating types D10	S+ x S-	1	2310	0,0792	0,0608	0,0832
		2	1629	0,0791	0,0586	0,0836
		3	2085	0,0795	0,0597	0,0829
		4	2661	0,0799	0,0608	0,0829
opposite mating types D7	*mcm1Δ+ x mcm1Δ-*		0	0	0	0
opposite mating types D7	*mcm1Δ+ x mcm1Δ- x Δmat*	1	832	0,0772	0,0608	0,0674
		2	723	0,0670	0,0544	0,0567
		3	439	0,0676	0,0554	0,0551
		4	529	0,0649	0,0544	0,0518
same mating types D7	*mcm1Δ+ x S+*	1	47	0,0342	0,0270	0,0231
		2	52	0,0354	0,0287	0,0236
		3	27	0,0378	0,0259	0,0297
		4	19	0,0336	0,0237	0,0288
same mating types D7	*mcm1Δ- x S-*	1	9	0,0215	0,0160	0,0185
		2	18	0,0250	0,0112	0,0343
		3	19	0,0312	0,0235	0,0243
		4	7	0,0338	0,0374	0,0096
same mating types D7	*mcm1Δ+ x mcm1Δ+*		0	0	0	0
opposite mating types D10	*mcm1Δ+ x mcm1Δ-*		0	0	0	0
opposite mating types D10	*mcm1Δ+ x mcm1Δ- x Δmat*	1	574	0,0566	0,0495	0,0384
		2	909	0,0600	0,0495	0,0460
		3	817	0,0549	0,0484	0,0409
		4	452	0,0569	0,0506	0,0379
same mating types D10	*mcm1Δ+ x S+*	1	79	0,0577	0,0353	0,0497
		2	89	0,0693	0,0591	0,0499
		3	66	0,0585	0,0324	0,0476
		4	63	0,0658	0,0512	0,0523
same mating types D10	*mcm1Δ- x S-*	1	27	0,0585	0,0395	0,0442
		2	26	0,0522	0,0305	0,0436
		3	44	0,0568	0,0311	0,0577
		4	26	0,0867	0,0672	0,0638
same mating types D10	*mcm1Δ+ x mcm1Δ+*		0	0	0	0
	pfd9	1	304	0,0524	0,0439	0,0450
		2	256	0,0722	0,0659	0,0520
		3	216	0,0564	0,0502	0,0451
		4	256	0,0472	0,0282	0,0472
	pdf23	1	214	0.0458	0.0188	0.0616
		2	194	0,0466	0,0188	0,0563
		3	249	0,0505	0,0188	0,0688
		4	349	0,0433	0,0188	0,0599
