# Supplementary material for: Mutations in Podospora anserina MCM1 and VelC Trigger Spontaneous Development of Barren Fruiting Bodies
Source: J Fungi (Basel). 2024 Jan 19;10(1):79. doi: 10.3390/jof10010079 (PMC10819945; doi:10.3390/jof10010079)

Figure S3. Sequences of the *VelC* homologues of *N. crassa* in strains ORS-SL6a and OR74A. The additional base in OR74A is boxed in white. Bottom, domain analysis using CD-search (https://www.ncbi.nlm.nih.gov/Structure/cdd/wrpsb.cgi) of the *VelC* homologue of ORS-SL6a showing that in this strain the velvet domain is complete.

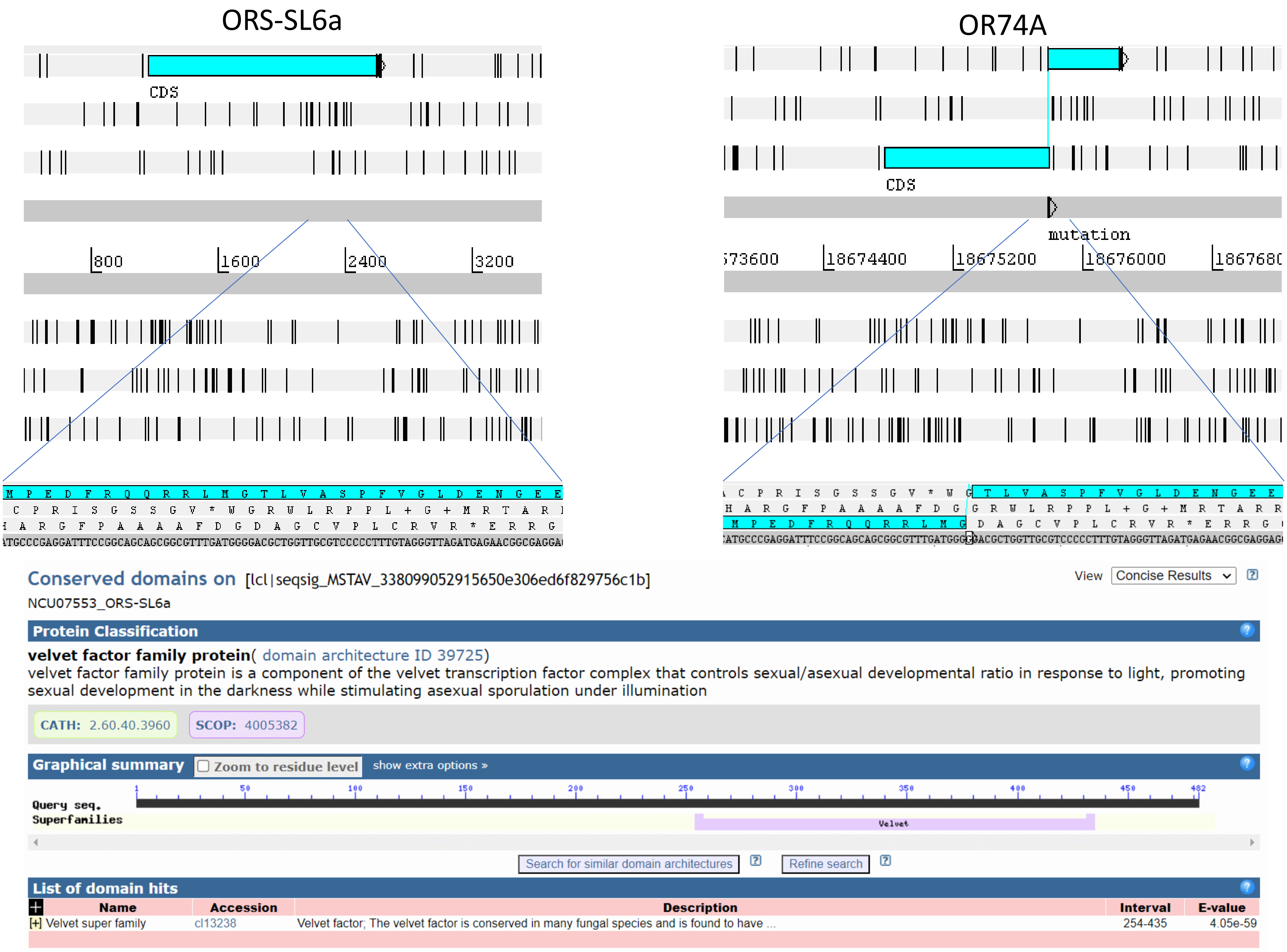

Supplement: Supplementary file 1 [file jof-10-00079-s001.zip › jof-2783895-supplementary1/Supporting Information Fig. S3.pdf]
