# Supplementary material for: Mutations in Podospora anserina MCM1 and VelC Trigger Spontaneous Development of Barren Fruiting Bodies
Source: J Fungi (Basel). 2024 Jan 19;10(1):79. doi: 10.3390/jof10010079 (PMC10819945; doi:10.3390/jof10010079)
Supplement: Supplementary file 1 [file jof-10-00079-s001.zip › jof-2783895-supplementary1/Supporting Information Table S1 revue.pdf]

Table S1. Primers used for *mcm1* (*Pa_1_19280), ste12* (*Pa-7-1730*) and *vacua* (*Pa-7-6330*) gene deletion, complementation and detection.

**Primer name**	**sequence**	**use**
Mcm1-1F	CGTGCCACCAACTCGCTATCC	Deletion and complementation of *MCM1*
Mcm-4R	AAAGAGTCTCAAGAACGACGG	Deletion and complementation of *MCM1*
Mk_Mcm-3F	CTTACCGCTGTTGAGATCCAG	Deletion of *MCM1*
Mk_Mcm-2R	CTATTTAACGACCCTGCCCTG	Deletion of *MCM1*
MCM1seq-F	AATACATGCCGCAATCCGGT	Verification construction of complementation of *MCM1*
MCM1seq-R	CGAGTTTCCATTGCCGACCG	Verification construction of complementation of MCM1
MCM1-V1	TGTCTGGTCCGTTCATATGGC	Detection of the *mcm1* deletion
MCM1-V2	CCCCAAGTACGTTGCTTGTCC	Detection of the *mcm1* deletion
pBC-For	CGCGCGTAATACGACTCA	Construction over-expressed *MCM1*
pBC-Rev	CGCGCAATTAACCCTCAC	Construction over-expressed *MCM1*
SurexMcm1Rev	GACGATATGGCGCGCTTAGACTGGACGGTTTACACAGGTGC	Construction over-expressed *MCM1*
TT_mcm1	GCACCTGTGTAAACGTCCAGTCTAAGCGCGCCATATCGTC	Construction over-expressed *MCM1*
AS4-1F	GCACGACCATTGGAACCAC	Construction MCM1-GFP
AS4_mcm1R	GCTGGTCAGTGATGTCGGCCATTTTGACGGTTTTGCGATC	Construction over-expressed MCM1 and MCM1-GFP
Mcm1_AS4_F	GATCGCAAAACCGTCAAAATGGCCGACATCACTGACCAGC	Construction over-expressed MCM1 and MCM1-GFP
Mcm1_fluo _R	CTCCTCGCCCTTGCTCACCATCGACTGGTGACCAGCGT	Construction MCM1-GFP
Fluo_97F	GCACGCTGGTCACCAGTCGATGGTGAGCAAGGGCGAGGAG	Construction MCM1-GFP
TT_216R	ACATCATTGTCCGAAGAGAGCC	Construction MCM1-GFP
vacuAfor	TCCTCTCACACCATGGCCCA	Complementation of *vacua*
vacuArev	CAAGAAAGCTCACCTCGCCA	Complementation of *vacua*
5'STE12	CCCACTCTGTCTCCTATCTAC	Deletion of *STE12*
3'STE12	GCGGATAAGAAGATGGTGAAGG	Deletion of *STE12*
5_CPC1_Mlu	GCGACGCGTCCGAGATGCGCCGCGTGC	Deletion of *STE12*
3_Ttrpc_Mlu	GTCACGCGTAGAGGATCCTCTAGCTA	Deletion of *STE12*
